# Safety and immunogenicity of plant-produced African horse sickness virus-like particles in horses

**DOI:** 10.1186/s13567-018-0600-4

**Published:** 2018-10-11

**Authors:** Susan J. Dennis, Martha M. O’Kennedy, Daria Rutkowska, Tsepo Tsekoa, Carina W. Lourens, Inga I. Hitzeroth, Ann E. Meyers, Edward P. Rybicki

**Affiliations:** 1 Biopharming Research Unit, Department of Molecular and Cell Biology, University of Cape Town, Rondebosch, Cape Town, 7701 South Africa; 2 Council for Scientific and Industrial Research (CSIR) Biosciences, Pretoria, 0001 South Africa; 3 Department of Veterinary Tropical Diseases, Faculty of Veterinary Sciences, University of Pretoria, Onderstepoort, Pretoria, 0110 South Africa; 4 Institute of Infectious Disease and Molecular Medicine, Faculty of Health Sciences, University of Cape Town, Observatory, Cape Town, 7925 South Africa

## Abstract

African horse sickness (AHS) is caused by multiple serotypes of the dsRNA AHSV and is a major scourge of domestic equids in Africa. While there are well established commercial live attenuated vaccines produced in South Africa, risks associated with these have encouraged attempts to develop new and safer recombinant vaccines. Previously, we reported on the immunogenicity of a plant-produced AHS serotype 5 virus-like particle (VLP) vaccine, which stimulated high titres of AHS serotype 5-specific neutralizing antibodies in guinea pigs. Here, we report a similar response to the vaccine in horses. This is the first report demonstrating the safety and immunogenicity of plant-produced AHS VLPs in horses.

**Electronic supplementary material:**

The online version of this article (10.1186/s13567-018-0600-4) contains supplementary material, which is available to authorized users.

## Introduction, methods and results

African horse sickness (AHS) is a highly infectious, notifiable viral disease which poses a substantial challenge to horse owners in sub-Saharan Africa. The aetiological agent, African horse sickness virus (AHSV), is an orbivirus of the family *Reoviridae*, and 9 different serotypes of the virus have been isolated [1, 2]. Immunization with live attenuated vaccines (LAVs) [3] has been the primary control strategy to date, but recent research has shown that vaccines of this nature carry the inherent risk of reverting to virulence, as well as the possibility of gene segment re-assortment between outbreak and vaccine strains [4]. Moreover, besides the inability to differentiate between natural and vaccine-induced immunity (non-DIVA), the LAV is restricted to use within southern Africa and this has significant implications for both the horse export industry as well as the equestrian sporting arena. There has long been demand for new, safe, efficacious and cost-effective DIVA vaccines, but although research demonstrating the efficacy of various alternative vaccine candidates has been published, none of these have yet been commercialized [5–8].

Virus-like particles (VLPs) are non-replicating protein assemblies identical in size and shape to the intact virus with which they share certain key features [9]. These VLPs are inherently safe and also DIVA compliant, as they contain no infectious genetic material nor any of the non-structural proteins. In a previous study, we demonstrated the *Agrobacterium tumefaciens*-mediated transient expression in *Nicotiana benthamiana* plants of the four structural proteins that make up the double-layered capsid of AHS serotype 5 viral particles (VP2, VP3, VP5 and VP7) [10]. Briefly, the gene sequences encoding the four capsid proteins of AHS 5 viral particles were codon-optimised for *Nicotiana* spp. translation and synthesized by GenScript Biotech Corporation, China. These genes were then cloned into the multiple cloning site of the pEAQ-*HT* plant vector [11] (obtained from G. Lomonossoff, John Innes Centre, UK) to yield four different constructs which were used to transform *Agrobacterium tumefaciens.* Transient expression of the AHSV proteins was achieved by *Agrobacterium*-mediated co-infiltration of the same plant with all four recombinant strains. Plants were incubated for 4 days to allow for recombinant protein expression and leaves subsequently harvested, ground up in 1× PBS containing 1× protease inhibitor (PI) cocktail P2714 (Sigma Life Science) and incubated overnight at 4 °C to allow for completion of VLP assembly. VLPs were purified by density gradient ultracentrifugation and TEM used to verify their similarity in structure to native AHS virions. Vaccination of guinea pigs with these AHS 5 VLPs proved to be completely safe and induced a high neutralizing antibody response in the animal model. The aim of the present study was to test the safety and immunogenicity of this VLP candidate vaccine in horses, and to consider ways in which the production platform could be scaled up in a cost-effective manner.

Eight horses were used in the study and these were all housed at the SA MRC facility in Cape Town. As the early history of these horses was not well documented, blood samples were drawn from the animals prior to vaccination (day 0) in order to ascertain the pre-vaccination immune status of the selected animals. AHSV protein VP7 is the group-specific antigen used in ELISA-based diagnostic tests [12]. Pre-vaccination sera were analysed by VP7 indirect ELISA at the Onderstepoort Veterinary Research Institute (ARC-OVI) in Pretoria, South Africa to determine prior exposure of the horses to AHS, either by vaccination with the currently used live attenuated vaccine (LAV) mixture produced by Onderstepoort Biological Products (OBP), or by natural infection. Furthermore, virus neutralization titres of the pre-vaccination serum samples against AHS were also determined. These tests were conducted at the Department of Veterinary Tropical Diseases at the University of Pretoria, as research using live AHS virus is not permitted in Cape Town, a small section of the Cape Town Metropole being the only area in South Africa where no cases of AHS have ever been known to occur.

The indirect ELISA results (Table 1) indicated that only three of the horses (horses 1, 2 and 5) were naïve prior to the study. The other five horses (horses 3, 4, 6, 7 and 8) all had positive VP7 iELISA scores most likely indicating prior vaccination with the AHS LAV. However, the virus neutralization titres (vnts) were negative for all eight horses and we therefore proceeded to use these horses in the safety and immunogenicity study.

**Table 1:** **VP7 indirect ELISA results and virus neutralising antibody titers (vnts) of the pre-vaccination sera**

Horse	iELISA—S/P %	vnt
Horse 1	Negative	Negative
Horse 2	Negative	Negative
Horse 3	83	Negative
Horse 4	34	Negative
Horse 5	Negative	Negative
Horse 6	35	Negative
Horse 7	39	Negative
Horse 8	44	Negative

Pre-vaccination sera of all 8 horses used in the experiment were assessed for AHSV antibodies (Ab) by indirect ELISA. High positive control serum pooled from vaccinated horses inoculated with live attenuated vaccine (bottle 1) obtained from Onderstepoort biological products (OBP), was used as a positive control. Sample/positive (S/P) percentage values less than 5.0 are classified as negative, S/P values greater than or equal to 5.0, but less than 10.0 are considered suspect, while S/P values greater than 10.0 are classified as positive for AHSV Ab. The horse sera were assayed for neutralisation capability against AHSV 5, AHSV 8 and AHSV 4 and all horses were shown to be negative against all three serotypes tested. Sheep serum from animals vaccinated with live AHS virus was used as a positive control.

The horses were vaccinated according to the schedule described in Table 2. Plant-produced AHS 5 VLPs used to vaccinate the horses in Group 1, were prepared and purified by iodixanol discontinuous density gradient ultracentrifugation of crude plant lysates as previously described [10], excepting that throughout this study, the wild-type AHSV 5 VP7 protein was expressed and incorporated into the VLPs rather than a mutated version used previously [13] as VLP yields were comparable. In order to obviate the need to use the expensive and sensitive equipment required for labour-intensive high-speed centrifugation and to eliminate the costly use of iodixanol, we also tested an alternative purification strategy to prepare the vaccine for the horses in Group 2. Briefly, a crude plant lysate containing AHS 5 VLPs was prepared as described previously [10], but using a bicine extraction buffer (20 mM NaCl, 50 mM bicine pH 8.4, 1 mM dithiothreitol (DTT) (Thermo Fisher Scientific) and 1× protease inhibitor (PI) cocktail P2714 (Sigma Life Science) instead of 1× PBS, as using a higher pH buffer seemed to stabilize the assembled VLPs. The plant extract was then filtered through a depth filter (Sartoclean GF sterile midicap, 3 µM + 8 µM) using a Masterflex console drive 4 peristaltic pump (Cole-Parmer Instrument Company). To further purify and concentrate the VLPs, the lysate was filtered through a 300 K Minimate™ TFF capsule (Pall Life Sciences) with the pressure not exceeding 2 Bar. The latter removes all proteins smaller than 300 K. The filtrate was washed with two volumes of bicine buffer lacking DTT. Finally, the extract was filter-sterilised through a 0.45 µM + 0.2 µM Sartobran 300 sterile capsule (Sartorius Stedim Biotech GmbH) using a peristaltic pump.

**Table 2:** **Vaccination schedule**

Group	Horses	Vaccine	Protein content	Dose and route of administration
1	Horse 1 Horse 2	Gradient purified AHS 5 VLPs	AHSV 5 VP2, VP3, VP5 and VP7	± 200 μg total protein in 5 mL PBS pH 7.4 with 5% Pet Gel A adjuvant, intra-muscular
2	Horse 3 Horse 4	Filtered AHS 5 VLPs	AHSV 5 VP2, VP3, VP5 and VP7	± 100 μg total protein in 5 mL bicine buffer pH 8.5 with 5% Pet Gel A adjuvant, intra-muscular
3	Horse 5 Horse 6	PBS	Nil	5 mL PBS pH 7.4 with 5% Pet Gel A adjuvant, intra-muscular
4	Horse 7 Horse 8	Bicine	Nil	5 mL bicine buffer pH 8.5 with 5% Pet Gel A adjuvant, intra-muscular

Horses were vaccinated with either gradient-purified (Group 1) or filtered (Group 2) plant-produced AHS 5 VLPs, or PBS (Group 3) or bicine buffer (Group 4), all adjuvanted with 5% Pet Gel A (SEPPIC, Paris, France) and administered by deep intra-muscular injection.

The two horses in Group 1 each received ± 200 μg adjuvanted (5% Pet Gel A) density gradient-purified AHS 5 VLPs, while the two horses in Group 2 each received ± 100 μg adjuvanted (5% Pet Gel A, SEPPIC) AHS 5 VLPs that had been purified by depth and tangential flow filtration. The reason for the difference in dose amounts was a result of the inability to achieve an equivalently high concentration of VLPs in the allowed dose volume using the alternative purification method. Groups 3 and 4 were the control vaccinated horses, receiving either adjuvanted (5% Pet Gel A, SEPPIC) PBS or bicine buffer. Upon vaccination, both Group 2 horses developed slight neck stiffness in the region where the vaccine was administered, but this subsided after 2 days. This was most likely due to the rather crude nature of the filtered vaccine preparation. No other side effects of the vaccinations were observed. Body temperatures were monitored daily and remained normal.

On day 27, blood samples were drawn from all the horses in order to monitor the immune status after the primary vaccination. The horses all then received a boost vaccination according to the same schedule described above. No side-effects were observed after the boost vaccination and the body temperatures of the horses continued to remain normal.

Blood samples from all 8 horses were drawn again on days 41, 69, 97, 125 and 153 post-vaccination and together with the pre-vaccination sera (day 0) were analysed for virus neutralization against the homologous AHSV 5 as well as against AHSV 8, as serological cross-protection has been shown in vitro between these two serotypes [14]. Virus neutralization titres against AHSV 4, for which no cross-protection by AHSV 5 has been shown, were also measured. Briefly, sera drawn at the various timepoints was serially diluted in minimal essential medium (MEM) containing 5% foetal calf serum and then mixed with 100 TCID_50_ AHSV. The presence of specific antibodies in the test serum inhibits the production of cytopathic effect (CPE) and the vnt is thus taken as the dilution at which 50% of the cells are infected.

**Figure 1:**
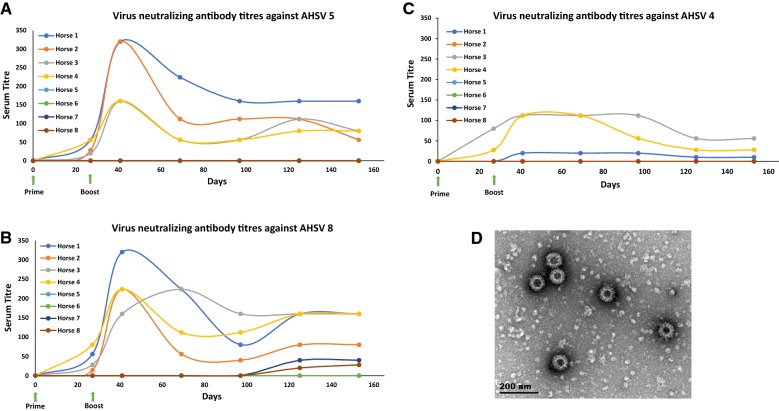
**Virus neutralising antibody titers of VLP-vaccinated and control horse sera against AHSV 5.** All horses were vaccinated on day 0 and received a boost vaccination on day 27. Pre- (day 0) and post- (days 27, 41, 69, 97, 125 and 153) virus neutralization titres (vnt) against serotypes 5 (**A**), 8 (**B**) and 4 (**C**) for all VLP- and control vaccinated horses were determined. The vnt is taken as the dilution at which 50% of the cells are infected. **D** Shows an electron-micrograph image of gradient-purified AHS 5 VLPs used to vaccinate horses 1 and 2. The scale bar is 200 nm.

The virus neutralization results are detailed in Additional file 1 and graphically depicted in Figure 1.

All AHS VLP-vaccinated horse sera neutralized both AHSV 5 and AHSV 8 to titres of 1:160–1:320 (log_10_ 2.2–2.5), while the AHSV positive control serum elicited a vnt of 1:320. Interestingly, neutralisation titres in serum from group 2 horses were notably lower than those from group 1 after the boost (Figure 1A). Furthermore, these sera also neutralized AHSV 4, although to a lesser extent (1:112 or log_10_ 2.0). Serum from the previously naïve horse 2 did not neutralise AHSV 4, although surprisingly, serum from horse 1, another horse naïve prior to vaccination, exhibited a low neutralization titre (1:20 or log_10_ 1.3) against AHSV 4 after boosting (Figure 1C). Although none of the four control horse sera neutralised AHSV 5, there was a very low neutralisation response measured when tested against ASHV 8 by the control horses in group 4 (Figure 1B).

Analysis of sera drawn on day 69 indicated a drop in viral neutralization titres in all four VLP-vaccinated horses against AHSV 5 and a subsequent plateauing effect for days 97, 125 and 153 post-vaccination. Interestingly, the vnt response against AHSV 4 in horses 3 and 4 which were not naïve prior to vaccination, plateaued for up to 97 and 69 days post-boost vaccination, respectively before declining. In the case of AHSV 8, both horses had titres which plateaued at a value of 1:160 (log_10_ 2.2.).

**Figure 2:**
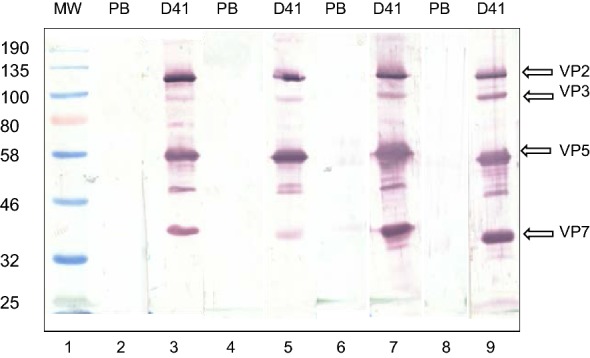
**Western blot analysis of pre- and post- (day 41) vaccination antisera.** Pre- and post- (day 41) vaccination antisera (1:5000 dilution) from horse 1 (lanes 2 and 3), horse 2 (lanes 4 and 5), horse 3 (lane 6 and 7) and horse 4 (lanes 8 and 9) were used to detect AHS 5 VLP proteins in a standard Western blot analysis. No AHSV proteins were detected in any of the pre-vaccination sera (PB) (lanes 2, 4, 6 and 8) but all four were detected in serum sampled at days 41 post-vaccination (D41) (lanes 3, 5, 7 and 9). Protein bands of AHS viral proteins VP2, VP3, VP5 and VP7 are indicated with arrows; colour pre-stained protein molecular weight marker (New England Biolabs, Massachusetts, USA) is in lane 1.

All pre- and post- (day 41) vaccination horse sera were also analysed by western blot against AHSV 5 capsid proteins separated by SDS-PAGE (Figure 2). Pre- and post-vaccination sera (1:5000) were used to probe a western blot of AHS 5 VLPs used in the initial inoculations. Strong signals for VP2, VP3, VP5 and VP7 were detected by post-vaccination sera from the VLP-vaccinated horses (lanes 3, 5, 7 and 9) but not by the control-vaccinated horses (results not shown) nor by the pre-vaccination sera (lanes 2, 4, 6 and 8) from any of the horses.

## Discussion

The results presented here show that all four horses vaccinated with the plant-produced AHS 5 VLPs, seroconverted after two doses of the vaccine. A maximum viral neutralization titre of 1:320 achieved for the two naïve horses (horses 1 and 2) was comparable to that generally obtained when inoculating horses with the AHS LAV [15]. The lower neutralization titres in serum from horses 3 and 4 could be a result of them receiving half the dose that horses 1 and 2 received. The results indicate that the vnt response in all VLP-vaccinated horses seemed to peak at 2 weeks post-final vaccination before declining, which concurs with the findings of others [6, 15]. The unexpected yet low vnts against AHSV 4 observed for the previously naïve horse 1, together with the late vnt response against AHSV 8 in both control horses 7 and 8 after 125 and 153 days, suggests to us that a vnt less than 1:40 could be regarded as a cut-off titre when drawing any conclusions with regard to the protective efficacy of the AHSV vaccine. A minimum cut-off titre of 16 has been stated for the commercially available LAV and Crafford et al. [16] suggest that protection of individual horses is likely to be effected by varying SNT titres. This should be an important point of consideration when evaluating future potential AHSV vaccine candidates.

Overall, the virus neutralization titres obtained, indicate that the plant-produced AHS 5 VLP vaccine may be at least as effective as the current LAV in protecting against AHSV 5, but without any of the associated risks of a live vaccine such as reversion to virulence or genetic re-assortment with field or vaccine strains. Furthermore, a similarly strong protective response against AHSV 8 was also elicited by all four horses, confirming the cross-protection between these two AHSV serotypes.

Sera from horses which were vaccinated with the VLPs prepared by depth and tangential flow filtration unexpectedly demonstrated strong neutralizing capability against AHSV 4. However, as could be seen from their positive iELISA scores prior to vaccination, they had obviously had some prior exposure to AHS and it is possible that immunization with the plant-produced VLPs boosted an earlier immune activation (one which would have included AHSV 8 and 4, but not AHSV 5 as the OBP LAV does not contain AHSV 5 [15]) resulting in an anamnestic response to the non-cross-reactive serotype 4. These results are in agreement with those published by Manning et al. [6], who demonstrated a similar immune boosting of an earlier vaccination with MVA VP2 (serotype 4) and MVA VP2 (serotype 9) when they subsequently vaccinated the same animals with the MVA VP2 (serotype 5) vaccine.

Aside from a transient local stiffness in the necks of the two horses that received the filtered plant extract, vaccination did not cause any adverse reactions, and all horses have remained healthy to date. These results demonstrate that the filtration strategy to purify the plant-produced AHS VLPs is a cost-effective alternative to purification by high speed density gradient ultracentrifugation, which makes this vaccine platform even more attractive. Further efforts are currently being made to refine this technique to produce cleaner, more concentrated vaccine samples and also to test the long-term stability of preparations.

We are currently exploring the possibility of initiating a challenge study using horses as well as investigating the production of VLPs of other AHS serotypes and/or the production of chimeric VLPs in a bid to meet the demand for a new, safer and economically viable AHS VLP vaccine. Furthermore, it is our intention to develop an iELISA test for one of the non-structural proteins so that our plant-produced VLP vaccine is DIVA compliant. Such a vaccine would address local concerns regarding the use of a live vaccine and would also serve as a potentially acceptable prophylactic or rapid response antidote in the wider international context.

## Additional file

**Additional file 1.** **Virus neutralising antibody titers and VP7 iELISA scores of VLP-vaccinated and control horse sera.** Pre- and post-vaccination sera were assayed for neutralisation capability against AHSV 5, AHSV 8 and AHSV 4. Sheep serum from animals vaccinated with live AHS virus was used as a positive control. The pre- and post-vaccination iELISA S/P scores of serum sampled prior to vaccination and 41 days post-vaccination are shown in the right hand column. High positive control sera pooled from vaccinated horses inoculated with live attenuated vaccine (bottle 1) obtained from OBP, was used as a positive control.

## Abbreviations

Ab: antibodies
AHS: African horse sickness
AHSV: African horse sickness virus
CPE: cytopathic effect
DAFF: Department of Agriculture, Forestry and Fisheries
DIVA: Differentiation between infected and vaccinated animals
DTT: dithiothreitol
FHS AEC: Faculty of Health Sciences Animal Ethics Committee
iELISA: indirect enzyme-linked immunosorbent assay
LAV: live attenuated virus
SAMRC ECRA: South African Medical Research Council Ethics Committee for Research on Animals
MEM: minimal essential medium
MVA: modified vaccinia Ankara
OBP: Onderstepoort Biological Products
OVI: Onderstepoort Veterinary Research Institute
PBS: phosphate-buffered saline
PI: protease inhibitor
SDS-PAGE: sodium dodecyl sulphate polyacrylamide gel electrophoresis
TCID_50_: median tissue culture infective dose
TFF: tangential flow filtration
VLP: virus-like particle
vnt: virus neutralization titre
VP: viral protein
