# Supplementary material for: Safety and immunogenicity of plant-produced African horse sickness virus-like particles in horses
Source: Vet Res. 2018 Oct 11;49:105. doi: 10.1186/s13567-018-0600-4 (PMC6389048; doi:10.1186/s13567-018-0600-4)
Supplement: Supplementary file 1 — Additional file 1. Virus neutralising antibody titers and VP7 iELISA scores of VLP-vaccinated and control horse sera. Pre- and post-vaccination sera were assayed for neutralisation capability against AHSV 5, AHSV 8 and AHSV 4. Sheep serum from animals vaccinated with live AHS virus was used as a positive control. The pre- and post-vaccination iELISA S/P scores of serum sampled prior to vaccination and 41 days post-vaccination are shown in the right hand column. High positive control sera pooled from vaccinated horses inoculated with live attenuated vaccine (bottle 1) obtained from OBP, was used as a positive control. [file 13567_2018_600_MOESM1_ESM.docx]

___________________________________________________________________________

Horse Day AHSV 4 AHSV 5 AHSV 8 iELISA

__________________________________________________________________________________

Horse 1 0 Negative Negative Negative Negative

27 Negative 1:56 1:40 -

41 1:20 1:320 1:320 120

69 1:20 1:224 1:224 -

97 1:20 1:160 1:80 -

125 1:10 1:160 1:160 -

153 1:10 1:160 1:160 -

Horse 2 0 Negative Negative Negative Negative

27 Negative 1:28 1:14 -

41 Negative 1:320 1:224 97

69 Negative 1:112 1:56 -

97 Negative 1:112 1:40 -

125 Negative 1:112 1:80 -

153 Negative 1:56 1:80 -

Horse 3 0 Negative Negative Negative 83

27 1:80 1:20 1:28 -

41 1:112 1:160 1:160 124

69 1:112 1:56 1:224 -

97 1:112 1:56 1:160 -

125 1:56 1:80 1:160 -

153 1:56 1:80 1:160 -

Horse 4 0 Negative Negative Negative 34

27 1:28 1:56 1:80 -

41 1:112 1:160 1:224 122

69 1:112 1:56 1:112 -

97 1:56 1:56 1:112 -

125 1:28 1:112 1:160 -

153 1:28 1:80 1:160 -

Horse 5 0 Negative Negative Negative Negative

27 Negative Negative Negative -

41 Negative Negative Negative Negative

69 Negative Negative Negative -

97 Negative Negative Negative -

125 Negative Negative Negative -

153 Negative Negative Negative -

Horse 6 0 Negative Negative Negative 35

27 Negative Negative Negative -

41 Negative Negative Negative 29

69 Negative Negative Negative -

97 Negative Negative Negative -

125 Negative Negative Negative -

153 Negative Negative Negative -

Horse 7 0 Negative Negative Negative 39

27 Negative Negative Negative -

41 Negative Negative Negative 124

69 Negative Negative Negative -

97 Negative Negative Negative -

125 Negative Negative 1:40 -

153 Negative Negative 1:40 -

Horse 8 0 Negative Negative Negative 44

27 Negative Negative Negative -

41 Negative Negative Negative 38

69 Negative Negative Negative -

97 Negative Negative Negative -

125 Negative Negative 1:20 -

153 Negative Negative 1:28 -

AHS (+) control >1:320 >1:320 >1:320 -

__________________________________________________________________________________
